# Models to predict injury, physical fitness failure and attrition in recruit training: a retrospective cohort study

**DOI:** 10.1186/s40779-020-00260-w

**Published:** 2020-06-03

**Authors:** Robin M. Orr, Bruce S. Cohen, Stephen C. Allison, Lakmini Bulathsinhala, Edward J. Zambraski, Mark Jaffrey

**Affiliations:** 1 Tactical Research Unit, Bond University, 2 Promethean Way, Gold Coast, Robina, QLD 4226 Australia; 2 United States Army Research Institute for Environmental Medicine, Natick, MA 01760 USA; 3 Defence Science and Technology Group, Port Melbourne, VIC 3207 Australia

**Keywords:** Military training, Predictive modelling, Risk management, Soldier

## Abstract

**Background:**

Attrition rate in new army recruits is higher than in incumbent troops. In the current study, we identified the risk factors for attrition due to injuries and physical fitness failure in recruit training. A variety of predictive models were attempted.

**Methods:**

This retrospective cohort included 19,769 Army soldiers of the Australian Defence Force receiving recruit training during a period from 2006 to 2011. Among them, 7692 reserve soldiers received a 28-day training course, and the remaining 12,077 full-time soldiers received an 80-day training course. Retrieved data included anthropometric measures, course-specific variables, injury, and physical fitness failure. Multivariate regression was used to develop a variety of models to predict the rate of attrition due to injuries and physical fitness failure. The area under the receiver operating characteristic curve was used to compare the performance of the models.

**Results:**

In the overall analysis that included both the 28-day and 80-day courses, the incidence of injury of any type was 27.8%. The 80-day course had a higher rate of injury if calculated per course (34.3% vs. 17.6% in the 28-day course), but lower number of injuries per person-year (1.56 vs. 2.29). Fitness test failure rate was significantly higher in the 28-day course (30.0% vs. 12.1%). The overall attrition rate was 5.2 and 5.0% in the 28-day and 80-day courses, respectively. Stress fracture was common in the 80-day course (*n* = 44) and rare in the 28-day course (*n* = 1). The areas under the receiver operating characteristic curves for the course-specific predictive models were relatively low (ranging from 0.51 to 0.69), consistent with “failed” to “poor” predictive accuracy. The course-combined models performed somewhat better than the course-specific models, with two models having AUC of 0.70 and 0.78, which are considered “fair” predictive accuracy.

**Conclusion:**

Attrition rate was similar between 28-day and 80-day courses. In comparison to the 80-day full course, the 28-day course had a lower rate of injury but a higher number of injuries per person-year and of fitness test failure. These findings suggest fitness level at the commencement of training is a critically important factor to consider when designing the course curriculum, particularly short courses.

## Background

Basic combat training prepares recruits for the demands of military life through physical training, combat training, and general military skill training [1, 2]. Recruits are typically drawn from the general population and commence training with varying levels of physical activity experience and fitness. The sudden increase in physical demands, the complexity of new physical tasks, and a reduced opportunity for recovery can lead to overtraining, and it is therefore not surprising that new recruits are at greater risk for musculoskeletal injury when compared to operational or incumbent soldiers [3–5].

Recruit injuries present a significant fiscal and personnel burden to military services, as injured recruits are up to 10 times less likely to complete recruit training [6]. In 1999, 59.0% of recruits in the Australian Army basic recruit training course were discharged as “medically unfit” [6]. The most common activities noted as causing injuries in Australian Regular Army recruits specifically were running (21.3%), marching (14.4%) and walking (7.0%). The two most frequently reported mechanisms of injury were overuse (33.4%) and overexertion (12.4%) [6].

As injuries delay graduation and increase attrition, both of which reduce the level of force readiness and availability for deployment [7–9], early identification of the trainees most at risk of training failure and musculoskeletal injuries is important. Screening levels have shown promising results, as apart from musculoskeletal injuries, poor physical fitness performance has been identified as a leading cause of attrition and delayed military recruit graduation in new recruits. A study by Pope et al. [6] predicting Australian Regular Army recruits’ risk of injury and attrition found that a score of level 3.5 on the 20-m multistage fitness test (MSFT), indicating low cardiovascular fitness, was associated with 14.2 times the risk of injury compared with a score of level 13.5. However, the remaining two components of the physical fitness assessment (push-ups and sit-ups) were poor predictors of injury [10]. The findings of this study are consistent with those from the general literature, which has likewise identified a lower level of fitness as a predictor of training failure in both general trainees (e.g., initial entry recruits and officers) [7, 11] and specialists (e.g., special forces trainees) [12]. Therefore, the aim of this study was to determine the best-fit analytic models for predicting injury, attrition, and failure to pass the final physical fitness test based on available predictor variables (including sex, age, and physical fitness assessment results) and common statistical methods.

## Methods

Data were retrospectively collected from two Australian Army recruit training courses during a period from 2006 to 2011: a 28-day reservist training course for Army reserve soldiers and an 80-day basic recruit training course for full-time soldiers. An identical set of entry standards were met in the two cohorts prior to training commencement. All training courses were conducted at a single site. Typically, a new cohort started the training every week throughout the year; on occasions, two cohorts started at the same time with a longer gap of two or 3 weeks between cohorts (e.g., over the December holiday period). The study was approval by the Australian Defence Human Research Ethics Committee (AF6185869), with all interagency cooperation documents, including the Cooperative Research and Development Agreement for Material Transfer and the Transfer Agreement for Existing Specimens or Data and the Data Transfer Agreements, signed.

### Injury data

Injury data were retrieved from the Defence Injury Prevention Program (DIPP) that included an injury surveillance tool for documentation of injuries in the 28- and 80-day recruit training courses. DIPP injury data included the type, location, activity during injury, action, severity and mechanism (cause). Data were collected at point-of-care facilities by medical staff, physiotherapists or physical training instructors (PTI) who closely monitored the program delivery and medical care for those sustaining injuries. All incidents that led to health care attention by a medical assistant, doctor or other health professional were documented in this system. This system has previously been shown to document up to 10 times as many of the injuries actually occurring as compared to the Australian Defence Force (ADF)’s Defence Work Health and Safety incident reporting system [13].

### Physical fitness assessments

#### Two fitness assessments were conducted

the Pre-enlistment Fitness Assessment (PFA) and the Recruit Fitness Assessment (RFA). The PFA consisted of push-ups, sit-ups and the 20-m MSFT and was conducted within 24 h of arrival at the training camp. If a recruit failed to meet the minimum standard (see Additional file 1: Table S1), a retest was scheduled for the next day. If a recruit failed the retest, he/she was removed from the training course and sent for remedial training in a separate platoon. The RFA was conducted in the last week of recruit training, with the same assessment items with the exception of the 20-m MSFT which was replaced with a 2.4 km run (see Additional file 1: Table S1). All assessments were conducted by Army PTI who undergo extensive and dedicated training in the application of these assessments, in accordance with formalized ADF protocols [14].

#### The push-up

The recruits began the test in the standard “up” position, with the body held in a straight line from head to heels and only hands and feet touching the floor. On command, the recruits bent their elbows and lowered themselves until their upper arms were parallel to the ground. The recruits then straightened their arms until they were back in the “up” position. Recruits were instructed to perform as many repetitions as possible in 2 min. Recruits could rest in the “up” position, with the hips raised or lowered; but had to return to the correct “up” position before commencing the next repetition. Prior to expiration of the 2 min, the test could be terminated when the recruits could no longer complete the push-up or were told to stop by the instructor due to safety concerns.

#### The sit-up

The sit-up assessment was conducted to an audio cadence of 1 repetition every 3 s. Recruits lay supine on a mat with their knees bent to 90 degrees and their feet on the floor. A partner could hold the feet secure to the floor if the recruit wished. Arms were straight with the palms resting on the front of the thighs and fingers extended. On the pre-recorded command “raise”, the recruits sat up until their wrists were over the top of their kneecaps and then lowered down at their own pace in preparation for the next repetition. The recruits’ feet and hips were not allowed to leave the floor, nor could they use their upper body to generate momentum. The test could be terminated when the recruit could no longer keep up with the audio recording or was told to stop by the instructor due to safety concerns.

#### The 20-m MSFT

Each level of the MSFT consisted of several 20-m shuttles. As the test progressed, the time allowed for each shuttle was reduced, so recruits were required to increase their running speed to complete the shuttle in the time allowed for each successive level. The test began at a speed just above a quick walking pace and increased to a full running speed at Level 7.5 (the minimum passing standard). If the recruit failed to place one foot on or over the 20-m line by the required auditory tone on two successive occasions or on three occasions cumulatively, the test was terminated, and the last successfully completed level was recorded.

#### The 2.4-km run

The recruit was required to complete a 2.4-km course as fast as possible. The course was conducted on a flat path with minimal undulating terrain. The time to complete the course was the measured outcome.

### Data preparation and analysis

The Australian Army data set included the DIPP injury data, performance data (initial, mid- and post-course fitness tests), and course start/completion dates. The data set also included basic anthropometric variables (age, height, weight) and course-specific variables (course number and military unit of assignment).

All data, character and numeric variable lengths, formats and properties were standardized. The resulting data were scrutinized to eliminate impossible or unlikely values that appeared to be due to data entry errors. Values outside the ranges of the accession standards (17 to 60 years of age, height > 152 cm, weight 42 to 150 kg, or body mass index (BMI) 18.5 to 32.9) were changed to missing values. Furthermore, data that could not accurately be read from paper-based entries or were coded as pass/fail were also changed to missing values.

Descriptive statistics were used to characterize the demographic and performance attributes of the recruits in the two recruit training courses and to quantify attrition and failure to pass the final physical fitness test battery. To determine the injury proportion (% of injuries), the number of personnel suffering one or more injuries was divided by the total number of recruits and multiplied by 100. The injury incidence rate (injuries per person-year) was calculated by dividing the number of recorded injuries by the length of the course and the population size and multiplying the result by 365 days.

Predictive models were developed for all targeted outcomes for each course separately and in combination. There were eight predictor variables available in the data set for the course-specific models at or near the beginning of training: age, sex, height, weight, BMI, initial 20-m MSFT, sit-ups, and push-ups. When models were established using both courses, course type (duration) became the 9th potential predictor variable. Given that the PFA tests tended to be assessed with recruits stopping upon attaining the minimum passing standards, these tests were also assessed for their predictive value after transformation to dichotomous pass/fail variables. Predicted outcomes in the models were any injury, overuse injury, stress fracture, neuromuscular injury, traumatic injury, attrition, and failure to pass the RFA. The injury subcategories for the prediction model were based on known injury concerns in military populations and areas of interest to research [1, 15]. A recruit was considered to have failed the RFA if the minimal passing standards were not met for one or more of the three individual tests.

Univariate analyses (unpaired *t*-tests for continuous variables and Chi-square test for sex) was used to select potential predictor variables for inclusion into the multivariate regression at a threshold of *P* < 0.20 [16]. Binary logistic regression was then used to filter the sets of potential predictor variables and to derive multivariate models to minimize redundant or substantially correlated predictors or predictors that did not contribute meaningfully to the multivariate prediction. The final logistic regression was performed using a forward conditional stepwise procedure with probability level at 0.05 for entry and 0.10 for removal. Logits for each recruit were computed from the final logistic regression equations and subsequently transformed into estimated probabilities of respective outcomes for every individual in the data set using the inherent transformation algorithm in the analytic software. The estimated probabilities for the outcomes, combined with observed occurrences or non-occurrence’s for injury and performance outcomes, were then used to construct receiver operating characteristic (ROC) curves. The areas under the curves (AUC) were computed to provide a general indication of performance risk for the models. A minimum AUC of 0.70 was expected for a model to have minimally acceptable predictive accuracy [17, 18].

Discriminant function analyses (DFA) were performed to construct potential alternative models for injuries and poor performance. Estimated probabilities of injury and performance outcomes were also computed based on the derived discriminant functions. ROC curves were constructed for these DFA-derived probability distributions as described above.

The model-based probability of each outcome was dichotomized into higher vs. lower probability using tables of coordinate points for the ROC curves and the Youden index [19]. The predictive performance of each model, using the maximum value in the distribution of Youden Indices as the cut score, was then characterized by calculating sensitivity, specificity, positive and negative predictive values, and positive and negative likelihood ratios. These predictive accuracy statistics were computed from 2 × 2 contingency tables containing frequency counts expressing numbers of recruits with true positive test results, false positive test results, true negative test results, and false negative test results. SPSS (version 23, IBM Corp, Armonk, NY, US) [20] was used for all statistical analyses.

## Results

The initial nonidentifiable data set contained results from 22,085 recruits completing their recruit training courses during the years 2006 to 2011. Data were deleted for all trainees in courses #202512 (*n =* 478) and #202513 (*n =* 172) as their data sets were incomplete. Data were also deleted for all entries assigned to the Training Support Company (*n =* 1666), given that these individuals were mostly those who were recycled through training because of injuries or other difficulties during initial training attempts or were staff members posted to assist in the training process. The final analysis included a total of 19,769 recruits (28-day course, *n =* 7692, 87.0% men; 80-day course, *n =* 12,077, 91.8% men). For both courses combined, the average recruit age was 22.2 ± 6.10 years.

The incidence of any type of injury during training was 27.8% in both courses combined. The proportion injured at least once was significantly lower in the 28-day course (17.6% vs. 34.3% in the 80-day course; *P* < 0.001). Accounting for exposure time, however, the any-type incidence rate was higher in the 28-day course (2.29 vs. 1.56 injuries per person-year in the 80-day course). Overall, women had a higher any-type injury incidence (43.3%) than men (26.0%) in both courses combined (*P* < 0.001); this held true even when exposure time was accounted for: 1.97 injuries per person-year for women and 1.19 injuries per person-year for men in both courses combined. When all-type injury incidence was stratified by both course type and sex, 31.0% of women were injured in the 28-day course (4.05 injuries per person-year) compared to only 15.5% of men (2.03 injuries per person-year). During the 80-day course, 55.7% of women experienced at least one type of injury (2.54 injuries per person-year), whereas only 32.4% of men were injured (1.48 injuries per person-year). A total of 45 stress fractures were recorded (13 in women; 32 in men), with an overall incidence of 0.2%; all but one occurred in the 80-day course, and all but one were lower extremity stress fractures. Attrition rate was not significantly different in the two courses: 5.2% in the 28-day course and 5.0% in the 80-day course (*P* = 0.66). The rate for failure to pass the final battery of fitness assessments (i.e., the RFA) was significantly higher in the 28-day course (30.0% vs. 12.1% in the 80-day course; *P* < 0.001). Detailed descriptive statistics are presented in Table 1.

**Table 1:** Descriptive statistics for the demographic, performance, and injury variables

Item	28-day Reservist Course		80-day Standard Course	
	Females	Males	Females	Males
^a^Age [year, mean ± SD (*n*)]	25.3 ± 7.4(1001)	24.1 ± 7.4(6686)	20.7 ± 4.8(990)	21.0 ± 4.7(11085)
^a^Height [cm, mean ± SD (*n*)]	166.2 ± 6.7(789)	179.0 ± 7.2(5444)	165.4 ± 6.2(845)	178.8 ± 7.0(9129)
^a^Weight [kg, mean ± SD (*n*)]	63.1 ± 8.1(810)	78.5 ± 10.8(5549)	63.3 ± 8.5(864)	78.1 ± 11.3(9371)
^a^Body mass index [kg/m^2^, mean ± SD (*n*)]	22.9 ± 2.4(787)	24.5 ± 2.9(5407)	23.1 ± 2.6(840)	24.4 ± 3.1(9099)
^a^Initial Push-ups [repetitions, mean ± SD (*n*)]	8.3 ± 2.3(535)	15.0 ± 1.0(3906)	8.6 ± 2.7(596)	15.0 ± 0.9(6413)
^a^Initial Push-ups Failure [No., (%, *n*)]	52 (9.7%, 535)	63 (0.9%, 3906)	50 (8.4%, 596)	82 (1.3%, 6413)
^a^Initial Sit-ups [repetitions, mean ± SD (*n*)]	44.8 ± 3.2(542)	45.0 ± 1.6(3907)	45.4 ± 6.0(607)	45.2 ± 2.7(6412)
^a^Initial Sit-ups failure [No., (%, *n*)]	14 (2.6%, 542)	21 (0.5%, 3907)	15 (2.5%, 607)	33 (0.5%, 6412)
^a^Initial Shuttle run [level, mean ± SD (*n*)]	7.3 ± 0.7(542)	7.5 ± 0.3(3911)	7.3 ± 0.9(608)	7.5 ± 0.2(6414)
^a^Initial Shuttle run failure [No., (%, *n*)]	100 (18.5%, 542)	80 (2.0%, 3911)	105 (17.3%, 608)	83 (1.3%, 6414)
Final Push-ups [repetitions, mean ± SD (n)]	25.2 ± 9.9(431)	43.2 ± 12.2(3302)	27.0 ± 8.1(513)	46.5 ± 11.8(5942)
Final Sit-ups [repetitions, mean ± SD (n)]	91.6 ± 17.2(439)	94.9 ± 12.4(3322)	96.6 ± 9.5(515)	97.6 ± 7.7(5953)
Final 2.4-km run [min, mean ± SD (n)]	11.7 ± 0.9(395)	10.3 ± 0.9(3111)	11.5 ± 0.7(495)	9.9 ± 0.8(5794)
^b^Any injury (%)	31.0% (311/1002)	15.5% (1040/6690)	55.7% (551/990)	32.4% (3587/11,087)
^b^Overuse injury (%)	18.1% (181/1002)	8.2% (550/6690)	45.9% (454/990)	21.1% (2337/11,087)
^b^Stress fracture (%)	0% (0/1002)	0.0% (1/6690)	1.3% (13/990)	0.3% (31/11,087)
^b^Neuromuscular injury (%)	28.9% (290/1002)	13.2% (880/6690)	49.9% (494/990)	28.4% (3152/11,087)
^b^Traumatic injury (%)	10.9% (109/1002)	6.1% (409/6690)	25.6% (256/990)	14.4% (1598/11,087)
^b^Attrition (%)	7.9% (27/341)	4.4% (51/1148)	4.5% (27/599)	5.0% (198/3945)
^b^Final RFA battery failure (%)	28.0% (112/400)	30.2% (953/3152)	8.9% (44/497)	12.4% (716/5797)

The results are presented as the mean ± SD for continuous-scale variables and as a number (% of total) for dichotomous outcomes (injuries, attrition, and failure on the final test battery). The percentage of total values for attrition and failure on the final test battery may be underestimated due to missing values. ^a^Potential predictor variables. ^b^Outcome variables for the predictive models. RFA. Recruit Fitness Assessment

Models derived with logistic regression and discriminant function analyses retained identical sets of predictor variables, and the resulting ROC curves were similar – with AUCs differing by no more than 5.0% between model derivation methods. Where there were differences, the logistic regression models generally performed slightly better than the other models. Therefore, the results of the logistic regression analyses are presented below.

### Course-specific predictive models

The models created for each course separately retained 1 to 6 predictors, with AUCs for the associated ROC curves ranging from 0.51 (predicting attrition in the 80-day course) to 0.69 (predicting stress fracture in the 80-day course). All models were statistically significant with omnibus tests of coefficients ≤0.028. All models showed acceptable goodness of fit, with the Hosmer-Lemeshow tests all being nonsignificant. However, the Nagelkerke *R*^2^ values were quite low, ranging from 0.01 to 0.10. The retained predictors, pseudo-R^2^ values, and AUCs are presented in Table 2 with the corresponding predictive equations in Table 3. Risk accuracy profiles for each course-specific model are presented in Additional file 2: Table S2 using cutoff values determined with the maximum Youden index.

**Table 2:** Retained predictors and associated areas under the ROC curves for the models for each course

Outcome	Course	Retained predictors	Nagelkerke *R*^2^	AUC
Any injury	28-day	Sex, Age, Init-SU, Init-shuttle^a^	0.06	0.63
	80-day	Sex, Age, BMI, Init-SU, Init-shuttle^a^	0.05	0.60
Overuse injury	28-day	Sex, Age, Init-SU, Init-shuttle^a^	0.05	0.63
	80-day	Sex, Age, BMI, Init-shuttle^a^	0.06	0.61
Stress fracture	28-day	(No model: only 1 stress fracture)	–	–
	80-day	Ht, Age, Init-shuttle	0.05	0.69
Neuromuscular injury	28-day	Sex, Age, Init-SU, Init-shuttle^a^	0.07	0.64
	80-day	Sex, Age, BMI, Init-shuttle^a^	0.05	0.60
Traumatic injury	28-day	Sex, Age	0.02	0.59
	80-day	Sex, Age, BMI, Init-SU^a^, Init-shuttle^a^	0.02	0.57
Attrition	28-day	Age	0.02	0.58
	80-day	Init-PU	0.01	0.51
Final BFA battery failure	28-day	Age, Wt, Init-PU^a^, Init-SU^a^, Init-shuttle^a^	0.10	0.64
	80-day	Ht, Wt, Init-PU^a^, Init-SU^a^, Init-shuttle^a^	0.07	0.64

*AUC* area under the ROC curve, *Init-PU* push-up repetitions obtained upon arrival at the basic training site, *Init-SU* sit-up repetitions obtained upon arrival at the basic training site, *Init-shuttle* shuttle run level obtained upon arrival at the basic training sitem, *Ht* height in cm, *Wt* weight in kg, *BMI* Body mass index in kg/m^2^, *BFA* Basic Fitness Assessment, ^a^failure. dichotomized pass/fail version of the predictor variable. -. No data

**Table 3:** Predictive equations for models created separately for each course

Outcome	Course	Equation
Any injury	28-day	Z = −3.805 + 0.928(Sex) + 0.034 (Age) + 0.049 (Init-SU) – 0.593(Init-shuttle^a^)
	80-day	Z = −1.877 + 0.871 (Sex) + 0.044 (Age) + 0.031 (BMI) + 0.020 (Init-SU) – 1.070 (Init-shuttle^a^)
Overuse injury	28-day	Z = −5.031 + 0.739(Sex) + 0.035(Age) + 0.064(Init-SU) – 0.845(Init-shuttle^a^)
	80-day	Z = − 1.621 + 1.032(Sex) + 0.046(Age) + 0.030(BMI) – 1.106(Init-shuttle^a^)
Stress fracture	28-day	(No model: only 1 stress fracture)
	80-day	Z = 6.184–0.042(Ht) + 0.077(Age) – 0.763(Init-shuttle)
Neuromuscular injury	28-day	Z = − 3.911 + 1.018(Sex) + 0.035(Age) + 0.046(Initial SU) – 0.637 (Init-shuttle^a^)
	80-day	Z = − 1.247 + 0.841(Sex) + 0.046(Age) + 0.027(BMI) – 0.957 (Init-shuttle^a^)
Traumatic injury	28-day	Z = − 3.087 + 0.642(Sex) + 0.028(Age)
	80-day	Z = −1.460 + 0.602(Sex) + 0.021(Age) + 0.032(BMI) – 0.746(Init-SU^a^) – 0.533(Init-shuttle^a^)
Attrition	28-day	Z = −3.943 + 0.040(Age)
	80-day	Z = −4.825 + 0.077(Init-PU)
Final BFA battery failure	28-day	Z = 2.263 + 0.024(Age) + 0.035(Wt) – 1.578(Init-PU^a^) – 3.549(Init-SU^a^) – 1.441(Init-shuttle^a^)
	80-day	Z = −8.775 + 0.033(Ht) + 0.025(Wt) + 0.032(Init-SU) – 1.455(Init-PU^a^) – 1.048(Init-shuttle^a^)

Z. logit from the logistic regression equation. Logits were converted to probabilities of the corresponding outcomes. Sex was coded 0 for females and 1 for males. Ht. height in cm; Wt. weight in kg; BMI. body mass index in kg/m^2^; Init-PU. push-up repetitions obtained upon arrival at the basic training site; Init-SU. sit-up repetitions obtained upon arrival at the basic training site; Init-shuttle. shuttle run level obtained upon arrival at the basic training site; ^a^failure. dichotomized pass/fail version of the predictor variable. BFA (failure) variables were coded 0 for passing and 1 for failure

### Predictive models including course type/duration as a predictor

**Fig. 1:**
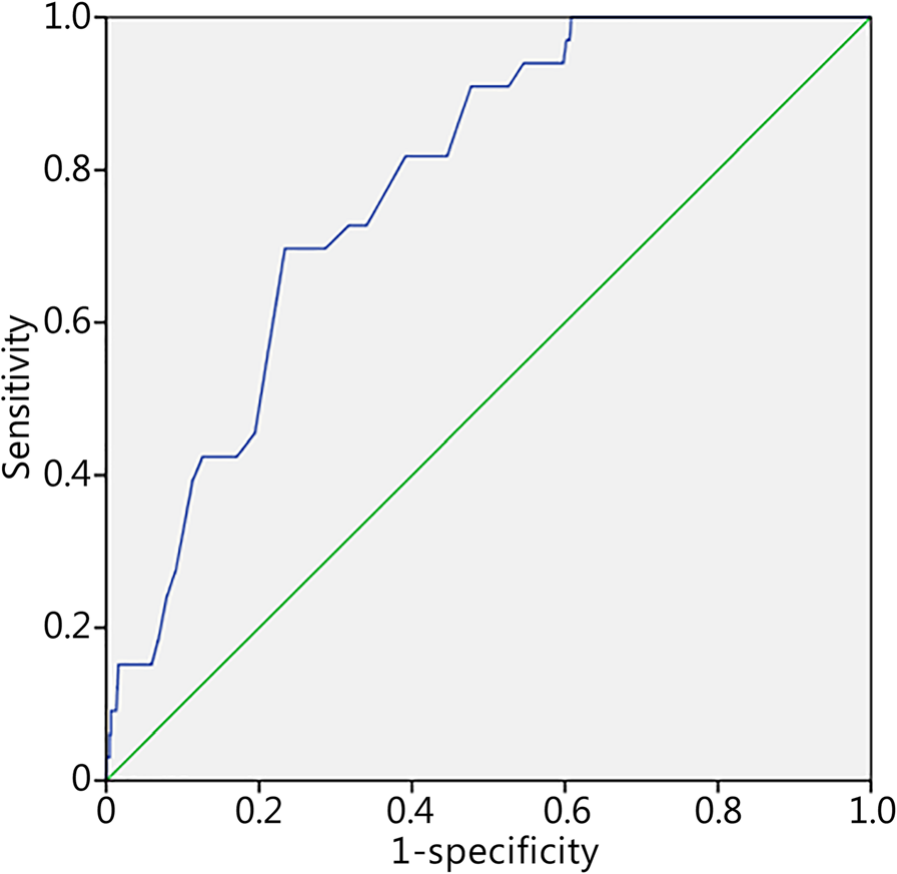
ROC curve for the model-based probability of stress fracture including course type as a predictor. Green line. Null line; Blue line. Individual cut off scores

**Fig. 2:**
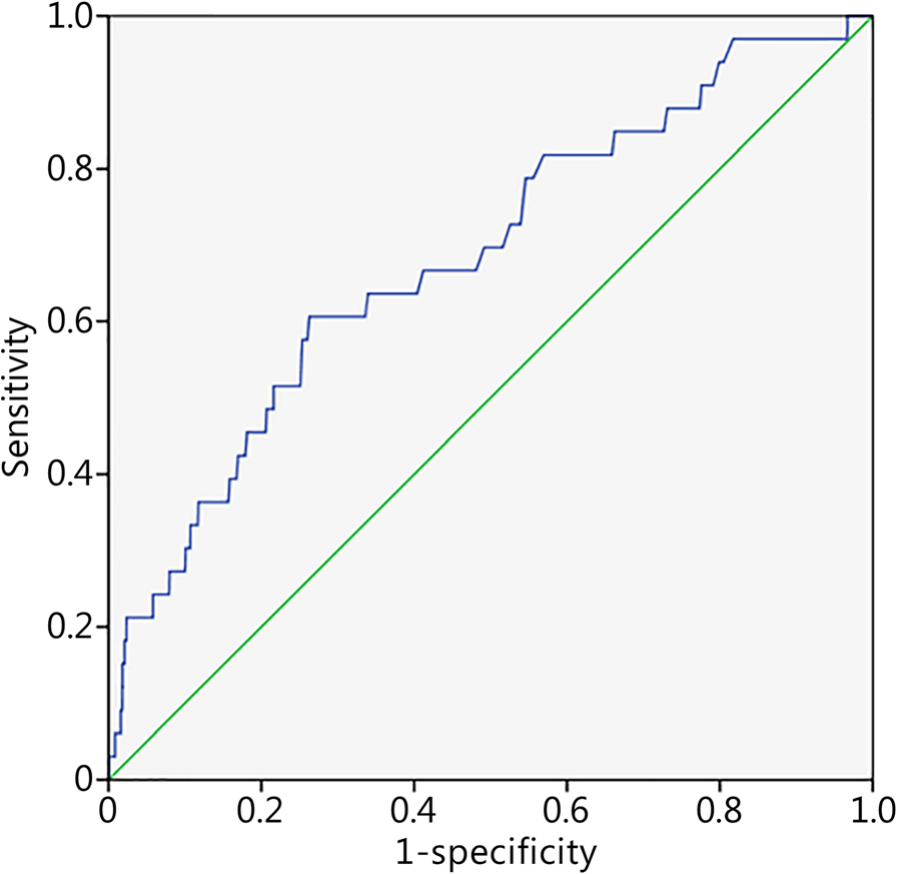
ROC curve for the model-based probability of stress fracture in the 80-day course. Green line. Null line; Blue line. Individual cut off scores

The models created with the combined data retained 2 to 7 predictors, with AUCs for the associated ROC curves ranging from 0.59 (predicting attrition) to 0.78 (predicting stress fracture). Course type was retained as a predictor in all models except for that for attrition. All models were statistically significant with omnibus tests of coefficients ≤0.001. All models showed acceptable goodness of fit, with the Hosmer-Lemeshow tests all being nonsignificant. Here, the Nagelkerke *R*^2^ values were relatively small. However, predictive performance was generally better with course-specific models, with the Nagelkerke *R*^2^ values ranging from 0.02 to 0.14, and two models (stress fracture prediction AUC = 0.78; failure of final physical fitness test battery prediction AUC = 0.70) in the “fair” category of risk accuracy. The retained predictors and AUCs are presented in Table 4, with the corresponding predictive equations provided in the additional files. Risk accuracy profiles for each course-combined model are presented in Additional file 3: Table S3, using outcome probability cutoff values determined with the maximum Youden index. Improvements in model performance when course type was added as a crude surrogate for exercise dose are illustrated in the ROC curves representing the prediction of stress fractures with (Fig. 1) and without (Fig. 2) course type as a predictor.

**Table 4:** Retained predictors and associated areas under the ROC curves for the models with both courses combined

Outcome	Retained Predictors	Nagelkerke *R*^2^	AUC
Any injury	Sex, Age, BMI, Init-SU, Init-Shuttle^a^, Course^b^	0.11	0.66
Overuse injury	Sex, Age, BMI, Init-SU, Init-Shuttle^a^, Course^b^	0.11	0.68
Stress fracture	Ht, Init-Shuttle, Course^b^	0.10	0.78
Neuromuscular injury	Sex, Age, BMI, Init-SU, Init-shuttle^a^, Course^b^	0.11	0.66
Traumatic injury	Sex, BMI, Init-SU, Init-shuttle, Init-SU^a^, Course^b^	0.05	0.63
Attrition	Age, Init-shuttle^a^	0.02	0.59
Final BFA battery failure	Ht, Wt, Age, Init-PU^a^, Init-SU^a^, Init-Shuttle^a^, Course^b^	0.14	0.70

*AUC* Area under the ROC curve, *Init-PU* Push-up repetitions obtained upon arrival at the basic training site, *Init-SU* Sit-up repetitions obtained upon arrival at the basic training site, *Init-shuttle* Shuttle run level obtained upon arrival at the basic training site, *Ht* Height in cm, *Wt* Weight in kg, *BMI* Body mass index in kg/m^2^, *BFA* Basic Fitness Assessment; ^a^Failure. Dichotomized pass/fail version of the predictor variable; ^b^Course number

## Discussion

In the current study, predictors for the injury models were generally consistent with earlier work identifying a female sex [9, 21, 22], greater height [21, 22], and poorer initial fitness levels [2, 21, 23] as the risk factors for injuries. The prevalence of injury in the current study (229 injuries per 100 person-years for the 80-day course and 156 injuries per 100 person-years for the 28-day course) is lower than that reported by Goodall et al. (316 injuries per 100 person-years of full-time equivalent service) [24], but higher than that reported in U.S. Army personnel (160 injuries per 100 person-years) [25]. A potential reason for these differences can be found in the paper by Pope and Orr [13], where the authors discuss differences in reporting practices and populations.

None of the models performed with good or excellent predictive accuracy, commonly defined as AUC values > 0.80 and > 0.90, respectively [17, 18]. The course-specific models performed poorly, with AUC values from the ROC curves mostly within ranges interpreted to represent predictive failure (AUC 0.50 to 0.60) or poor discriminative prediction ability (AUC 0.60 to 0.70) [17, 18]. However, two models created with data from both courses combined, which allowed for the inclusion of “course type (duration)” as a predictor in the models, performed somewhat better, i.e., in the minimally acceptable or “fair” discrimination range (AUC 0.70 to 0.80) [17, 18]. Overall, predictive accuracy, as reflected in the AUC values, was consistent with that in previous work. For example, George et al. [26] found an AUC of 0.64 for a logistic regression model used to predict first episodes of lower back pain in soldiers undergoing combat medic training. Moran et al. [27] reported an AUC of 0.77 for a 5-predictor logistic regression model for predicting stress fractures in female recruits during basic training. However, those authors were able to obtain an AUC of 0.91 with an unwieldy 20-predictor model for the same outcome; none of the models in the current study had comparable AUCs.

The cumulative volume of physical activity and the exercise dose were substantially greater in the 80-day course than in the 28-day course. The fact that all but 1 of the 45 stress fractures occurred in the 80-day course reflects longer period of chronic loading in our opinion. However, with the requirement of similar skill development between courses, it is acknowledged that the shorter 28-day course may have been more intensive (e.g., fewer personal administration periods) than the 80-day course. The improvement in predictive performance of the combined-course models suggests that predictive models should capture physical activity dose if possible to yield levels of predictive accuracy that would make the models useful for identifying recruits at high vs. low risk of adverse training outcomes. As a surrogate for physical activity, course type (duration) (short vs. long) was retained as a predictor in every model derived from both courses combined, except for the model predicting attrition, which was essentially the same in both courses.

The failure rate for the final battery of RFAs was higher (30.0%) in the 28-day course than in the 80-day course (12.1%). This may be attributed to the cumulative volume of training (physical activity and physical activity dose). Most exercise training programs recommend up to 12 weeks to achieve noticeable changes in the aerobic, muscular strength and endurance fitness components [28, 29]. Hence, the 28-day (shorter duration) course may not adequately physically prepare some recruits for their future training courses (e.g., infantry training or artillery training).

The RFA failure rate was high in the 28-day course in comparison to the U.S. Army Physical Fitness Test (APFT) failure rate, which is approximately 15% [30], whereas the 80-day course levels were commensurate if not slightly lower (12.1%). Unlike the RFA, the APFT is age-graded. Furthermore, there are differences in sit-up protocols, and the run is slightly longer for the APFT at 3.2 km (2 mi). Considering this, for the age group range of 22–26 years, the APFT push-up requirements are 31 (RFA = 35) repetitions for male recruits and 11 (RFA = 18) repetitions for female recruits, allowing for some comparison of this variable.

The selection of cutoff values for high vs. low risk for adverse outcomes must be made with a balance between falsely identifying a recruit as high-risk (a false positive prediction) or as low-risk (a false negative prediction). Cutoff values yielding high sensitivity and high negative predictive values protect preferentially against false negatives, i.e., relatively few recruits would be falsely identified as low-risk. Alternately, cutoffs yielding high specificity and high positive predictive values protect preferentially against false positives, i.e., relatively few recruits would be falsely identified as high-risk. As such, predictive or diagnostic models typically demonstrate a sensitivity-specificity tradeoff: selecting a cut score yielding high sensitivity will yield relatively low specificity and vice versa [31]. This tradeoff was evident in the models derived in this study. The implications for these findings are that the command elements of the Army could adjust this sensitivity-specificity equation balance to meet recruit, and hence combat force size, thresholds.

The results from this study are likely influenced by multiple important limitations. Although extensive efforts were made to document injury data, it is possible that some recruits failed to report injuries. Individual recruits who are highly motivated to graduate from basic training may conceal injuries that will surface in subsequent training. As an example, nearly two-thirds (64.0%) of all U.S. Army trainees injured during initial entry training had symptoms of musculoskeletal injuries (SMSKI) that they did not report to leadership or a medical provider. The most common reasons selected for not reporting their SMSKI (i.e., not seeking medical care) included “I wanted to graduate on time” and “I wanted to avoid a profile (*i.e.*, medical limitation for job-related tasks)” [32]. Future studies may be able to document more complete injury data by following recruits for injuries that are reported early in training subsequent to basic military training. Many potential predictors of interest, such as prior injuries [2], smoking status [2, 23], race/ethnicity [22, 33], self-reports of physical activity levels prior to training [2, 34], exercise dose [2, 35], joint flexibility [2, 36], age of running shoes [37], and individual biomechanical attributes (e.g., valgus knees / q-angle greater than 15 degrees, dynamic pes planus, pes cavus, restricted ankle dorsiflexion) [15, 23, 38], were not available in this data set. Likewise, known risk factors for attrition, such as physical or sexual abuse [8] and mental health history [8], were not available for analysis in this study. The predictive accuracy of the models may have improved meaningfully if these additional variables had been available. Future prospective studies to derive predictive models should include the full spectrum of known and suspected risk factors for negative training outcomes.

The results from this study suggest that the inclusion of physical activity dose in predictive models may yield higher levels of predictive accuracy. Furthermore, measurement or estimation of the biomechanical attributes of recruits (where feasible) should be included in future predictive models, as it has been shown to improve the prediction of injuries during military training [23]. It is possible that complex modelling methods exploring nonlinear relationships among injuries, poor physical fitness, exercise dose, and individual biomechanical factors may yield greater risk accuracy than can be obtained with common statistical procedures, such as those employed in this study.

## Conclusions

The models performed with levels of prognostic accuracy considered ‘failing’ to ‘fair’ in identifying factors capable of predicting the probability of RFA failure and attrition in this population of Army recruits. As such, the factors associated with RFA failure and attrition identified in the models proposed, including age, sex, height, weight, and initial fitness test scores, should be viewed with caution. However, the findings regarding the differences between the two training courses and between the sexes, and the factors predictive of failure and attrition can still be used to inform future physical training and injury mitigation strategies for both courses. Though there were similar attrition rates between the courses, the 28-day course had a higher fitness test failure rate than the 80-day course. Women, who are known to have a lower level of fitness in general, had a higher any-type injury incidence than men, even when accounting for exposure time. As such, initial levels of fitness at the commencement of training (especially for the 28-day course) may be of paramount importance in designing interventions to mitigate fitness test failure and injury.

## Supplementary information

**Additional file 1: Table S1.** Passing standards for recruit pre-enlistment fitness assessment and recruit fitness assessment (Australian Army 2009).

**Additional file 2: Table S2.** Prognostic accuracy profiles for the models created separately for each course, with cut scores determined by the probabilities of the outcome associated with the maximum Youden index values.

**Additional file 3: Table S3.** Prognostic accuracy profiles for models created from both courses combined, with cut scores determined by probabilities of the outcome associated with maximum Youden index values.

## Data Availability

As the data are drawn from a military population, the data and materials will only be made available upon a formal specific request made to the corresponding author who will seek approval from the relevant agencies. A formal request will not infer approval.

## Abbreviations

ADF: Australian Defence Force
APFT: Army Physical Fitness Test
AUC: Area under the curve
BMI: Body mass index
DFA: Discriminant function analyses
DIPP: Defence Injury Prevention Program
MSFT: Multistage fitness test
PFA: Pre-enlistment Fitness Assessment
PTI: Physical training instructors
RFA: Recruit Fitness Assessment
ROC: Receiver operating characteristic curve
SMSKI: Symptoms of musculoskeletal injuries
