# Supplementary material for: Models to predict injury, physical fitness failure and attrition in recruit training: a retrospective cohort study
Source: Mil Med Res. 2020 Jun 3;7:26. doi: 10.1186/s40779-020-00260-w (PMC7271478; doi:10.1186/s40779-020-00260-w)
Supplement: Supplementary file 1 — Additional file 1: Table S1. Passing standards for recruit pre-enlistment fitness assessment and recruit fitness assessment (Australian Army 2009). [file 40779_2020_260_MOESM1_ESM.docx]

**Additional files Table 1**  Passing standards for recruit pre-enlistment fitness assessment and recruit fitness assessment (Australian Army 2009).

| Items | Standards | |
| --- | --- | --- |
|  | Males | Females |
| Pre-enlistment assessment pass mark | |  |
| Push-ups (repetitions) | 15 | 8 |
| Sit-ups (repetitions) | 45 | 45 |
| 20, PSRT (Level) | 7.5 | 7.5 |
| Recruit fitness assessment pass mark | |  |
| Push-ups (repetitions) | 35 | 18 |
| Sit-ups (repetitions) | 70 | 70 |
| 2.4 km Run | 11:18 min | 13:30 min |
