# Supplementary material for: Models to predict injury, physical fitness failure and attrition in recruit training: a retrospective cohort study
Source: Mil Med Res. 2020 Jun 3;7:26. doi: 10.1186/s40779-020-00260-w (PMC7271478; doi:10.1186/s40779-020-00260-w)
Supplement: Supplementary file 2 — Additional file 2: Table S2. Prognostic accuracy profiles for the models created separately for each course, with cut scores determined by the probabilities of the outcome associated with the maximum Youden index values. [file 40779_2020_260_MOESM2_ESM.docx]

**Additional files Table 2** Prognostic accuracy profiles for the models created separately for each course, with cut scores determined by the probabilities of the outcome associated with the maximum Youden index values

| Model | Course | Cut score | Sn | Sp | PLR | NLR | PPV | NPV |
| --- | --- | --- | --- | --- | --- | --- | --- | --- |
| Any injury | 28-day | ≥ 21.8% | 0.51 | 0.69 | 1.68 | 0.71 | 0.33 | 0.83 |
|  | 80-day | ≥ 45.2% | 0.35 | 0.79 | 1.70 | 0.82 | 0.57 | 0.62 |
| Overuse injury | 28-day | ≥ 11.6% | 0.54 | 0.68 | 1.68 | 0.68 | 0.19 | 0.92 |
|  | 80-day | ≥ 30.7% | 0.38 | 0.80 | 1.88 | 0.78 | 0.44 | 0.76 |
| Stress fracture | 28-day | - | - | - | - | - | - | - |
|  | 80-day | ≥ 0.6% | 0.61 | 0.74 | 2.31 | 0.53 | 0.01 | 1.00 |
| Neuromuscular injury | 28-day | ≥ 19.4% | 0.48 | 0.74 | 1.85 | 0.70 | 0.31 | 0.85 |
|  | 80-day | ≥ 40.1% | 0.36 | 0.79 | 1.69 | 0.81 | 0.52 | 0.66 |
| Traumatic injury | 28-day | ≥ 8.2% | 0.56 | 0.59 | 1.36 | 0.75 | 0.09 | 0.95 |
|  | 80-day | ≥ 18.9% | 0.49 | 0.62 | 1.29 | 0.82 | 0.24 | 0.84 |
| Attrition | 28-day | ≥ 5.5% | 0.49 | 0.69 | 1.57 | 0.74 | 0.08 | 0.96 |
|  | 80-day | ≥ 1.8% | 0.92 | 0.11 | 1.04 | 0.68 | 0.02 | 0.98 |
| Final BFA battery failure | 28-day | ≥ 31.1% | 0.50 | 0.74 | 1.89 | 0.68 | 0.44 | 0.78 |
|  | 80-day | > 12.7% | 0.53 | 0.69 | 1.69 | 0.69 | 0.19 | 0.91 |

Cut scores are in units of probability of the outcome, as transformed from logits computed from the logistic regression models. Sn. sensitivity; Sp. specificity; PLR. positive likelihood ratio; NLR. negative likelihood ratio; PPV. positive predictive value; NPV. negative predictive value; BFA. Basic Fitness Assessment. -. No data.
