# Supplementary material for: Models to predict injury, physical fitness failure and attrition in recruit training: a retrospective cohort study
Source: Mil Med Res. 2020 Jun 3;7:26. doi: 10.1186/s40779-020-00260-w (PMC7271478; doi:10.1186/s40779-020-00260-w)
Supplement: Supplementary file 3 — Additional file 3: Table S3. Prognostic accuracy profiles for models created from both courses combined, with cut scores determined by probabilities of the outcome associated with maximum Youden index values. [file 40779_2020_260_MOESM3_ESM.docx]

Additional files Table 3 Prognostic accuracy profiles for models created from both courses combined, with cut scores determined by probabilities of the outcome associated with maximum Youden index values

| Model | Cut score | Sn | Sp | PLR | NLR | PPV | NPV |
| --- | --- | --- | --- | --- | --- | --- | --- |
| Any injury | ≥35.2% | 0.77 | 0.46 | 1.43 | 0.50 | 0.44 | 0.78 |
| Overuse injury | ≥ 23.6% | 0.71 | 0.54 | 1.54 | 0.54 | 0.32 | 0.86 |
| Stress fracture | ≥ 0.5% | 0.70 | 0.77 | 2.98 | 0.40 | 0.01 | 1.00 |
| Neuromuscular injury | ≥ 31.5% | 0.74 | 0.50 | 1.46 | 0.53 | 0.40 | 0.81 |
| Traumatic injury | ≥ 15.7% | 0.74 | 0.45 | 1.35 | 0.58 | 0.20 | 0.90 |
| Attrition | ≥ 3.4% | 0.36 | 0.80 | 1.82 | 0.80 | 0.06 | 0.98 |
| Final BFA battery failure | ≥ 21.1% | 0.57 | 0.73 | 2.11 | 0.59 | 0.32 | 0.88 |

Cut scores are in units of probability of the outcome, as transformed from logits computed from the logistic regression models. Sn. Sensitivity; Sp. Specificity; PLR. Positive Likelihood Ratio; NLR. Negative Likelihood Ratio; PPV. Positive Predictive Value; NPV. Negative Predictive Value. BFA. Basic Fitness Assessment.
